# Self-Treatment Practices for Perceived Symptoms of Malaria in Ethiopia

**DOI:** 10.7759/cureus.9359

**Published:** 2020-07-23

**Authors:** Vitaley Kovalev, Michael L Wells

**Affiliations:** 1 Acute Care Surgery, California Hospital Medical Center, Los Angeles, USA; 2 Basic Medical Sciences, College of Osteopathic Medicine of the Pacific, Western University of Health Sciences, Pomona, USA; 3 Cardiothoracic Surgery, University of Colorado Hospital, Colorado, USA

**Keywords:** malaria, ethiopia, treatment

## Abstract

One of the most significant public health issues faced in Ethiopia is malaria. The most influential problem of this public health issue is overcoming barriers of having proper access to professional care and treatment. This study aims to elucidate the self-interventions undertaken by individuals when they perceive symptoms of malaria in a family-member prior to, or instead of, seeking care at a healthcare facility. Our study found that the prevalence of self-medication for malaria in the Wirtu Yedi kebele is 37.3%. Almost all individuals eventually sought treatment for malaria at the healthcare facility. More than half did so in less than one day. When self-treatment was used, there was a wide array of self-medication practices used, including modern medications not prescribed by a healthcare professional, herbs, and non-pharmacological measures. The more commonly used medications were chloroquine and Coartem® (artemether and lumefantrine). Most reported obtaining these medications at a drug outlet store without a prescription and prior to seeking care at a health center. Of the various herbs reported that were used to self treat malaria the most commonly used were garlic, ginger, and harmaguse. The use of herbs was found to be less common than the use of modern medication. Of the non-pharmacological interventions, rituals were the most prevalent.

## Introduction

One of the most significant public health issues faced in Ethiopia is malaria. In 2011, three-quarters of the Ethiopian territory was considered endemic for malaria, putting more than 60 million people (or about 60% of the total population) at-risk [1]. The most influential problem of this public health issue is overcoming barriers of having proper access to professional care and treatment. Malaria, caused by the parasite *Plasmodium*, is transmitted by the bite of infected female anopheline mosquitoes. During the mosquitoes feeding, sporozoites are introduced into the human’s bloodstream, which circulates to the liver. Its life cycle consists of multiplying in the liver and releasing merozoites that travel systemically to infect red blood cells that ultimately result in hemolysis of the cells [2]. This pathology can ultimately result in death if not adequately and timely treated. Of the various malarial species in Ethiopia, 85% are found to be *P. falciparum* [3].

The role of a professional healthcare facility in treating malaria is crucial to help reduce morbidity and mortality. This is because prompt and accurate parasitological confirmation of malaria is essential for effective disease management and treatment. Currently, diagnostic tools, such as microscopy and rapid diagnostic tests, help to identify the presence of malaria infection. It is important for the specific type of species to be identified due to the development of resistance to some antimalarial drugs to certain *Plasmodium *species and, therefore, will influence what course of treatment is most appropriate.

This public health issue dramatically increases and becomes more difficult to overcome when considering various rural areas within Ethiopia. This is because there is less access to professional healthcare, and as a result, many seek other alternative measures instead of relying on a structured healthcare system. Out of an estimate of 9 million cases per year, only 4 million will be actually treated in a structured healthcare facility [4]. Studies have shown that the other 5 million utilize other resources to help treat the disease. Of these rural areas found in Ethiopia, one area that continually is found to be at risk is the town of Asendabo. For instance, a study done in various rural kebeles in Asendabo showed that approximately 39.2% utilize self-medication practices of people who got sick with malaria [5]. The practices that were utilized included obtaining modern drugs from alternative sources or treatments using herbs, non-pharmacological measures (such as holy water or exercise), and 9.8% took no action at all [5].

An additional barrier that patients must overcome is having the knowledge of when it is most appropriate to seek professional medical treatment due to symptoms of malaria. This is because commonly presenting symptoms of malaria are constitutional and, therefore, can be hard to distinguish from other benign etiologies, such as the common cold. Normal prodrome symptoms of malaria include headache, fatigue, fever, and/or malaise. Other common presenting symptoms include myalgias, arthralgias, cough, chest pain, abdominal pain, anorexia, nausea, vomiting, and/or diarrhea. Thus, patients may be more likely to utilize treatment methods that concentrate on treating the symptoms of malaria and not actually treat the infectious process.

Thus far, there has not been any research conducted on specific interventions of self-treatments used at home prior to or instead of seeking professional healthcare treatment in the various kebeles of Asendabo. The town of Asendabo is made up of three kebeles, Haro Gibe, Abdi Gudina, and Wirtu Yedi. The health center located in Asendabo is responsible for nine total catchment areas of Asendabo. This study aims to recognize the actual types of self-interventions undertaken by individuals when they perceive they have symptoms related to malaria. It will specifically identify methods undertaken by the Wirtu Yedi community and the influence of the Asendabo health center in their decision-making process. Furthermore, it shows the timeline of when an individual in this population recognizes when it is most appropriate to seek professional treatment once symptoms occur. This study will also address various correlations of self-treatment practices as related to the demographics within the population of this kebele.

## Materials and methods

The study area was chosen within Jimma University’s field research project region. The specific region chosen was the Wirtu Yedi kebele (the smallest administrative unit in Ethiopia) of the Asendabo town, which is a capital of the Omo Nado Woreda (or district), which is part of Jimma zone, in the Oromia regional state (the largest administrative unit of Ethiopia). The town of Asendabo is located 258 km away from Addis Ababa and 55 km north of the Jimma City. The Wirtu Yedi kebele is one of three kebeles comprising the Asendabo area. This area’s population consisted of 3,800 people. The population of Witru Yedi has access to a healthcare center, which is the designated healthcare facility of the town of Asendabo.

A community-based cross-sectional study was used to assess the caregivers’ choice of self-treatment of symptoms perceived to be indicative of a malaria infection prior to seeking professional medical treatment at a regional health facility. Caretakers of the household were approached and asked to participate in the study survey. Inclusion criteria for recruitment to participate in the study were caretakers who believed they have had any person sick with malaria in their household. Persons interviewed were excluded if they believed to have never had anyone sick with malaria in their household. They were also excluded if they have had anyone sick with malaria in their household but did not choose to use any method of self-treatment prior to or instead of seeking care at a medical facility. Persons who claimed that they did not “know” what malaria was were chosen to be included in the study because of the concern that “knowledge” of malaria may be interpreted in a multitude of ways, including the knowledge of physiology, which is not a necessary prerequisite for an individual to be able to make decisions regarding treatment. The sample size was calculated with the assumption of a 39% prevalence of self-medication in Asendabo with a confidence interval (CI) of 95%. The sample size was calculated at n = 366.

Information regarding the most common methods of self-treatment for malaria was gathered from previous studies, our key informant at Asendabo Tamiru Mekonnen, and interviews with pharmacy workers in Jimma [5-6]. A structured questionnaire was developed based on the objectives of the study (Appendix A). The questionnaire was developed in English and then translated to Afan Oromo, a local language with the help of Tamiru Mekonnen. The questionnaire was then translated back to English to check for consistency. The first part of the questionnaire focused on selecting relevant respondents for the study, the second part focused on eliciting the type and methods of self-treatment used, and the last part comprised the demographic portion. The questionnaire contained 25 questions. Questions 1 to 3 provided unique identifiers for each questionnaire with the house number being providing the only traceable information back to the household interviewed. Questions 4 through 11 were designed to select respondents relevant to the study based on the exclusion and inclusion criteria. Question 14 asked about self-treatment methods based on commonly used herbs in the area, non-pharmacological methods, and pharmacological treatments. For the pharmacological medication portion of the questionnaire, the data collectors were provided with a sample of pills of each of the three medications used for the treatment of malaria in the region, chloroquine, Coartem, and quinine. These samples were used for the visual identification of these medications. Questions 13 through 16 collected information about the proper use and source of pharmacological medications. Questions 17 and 18 asked about the individual’s perception of self-treatment outcomes and barriers to seeking care at a healthcare facility. Questions 18 through 25 collected demographic information which included possible confounding factors based on the literature review [7]. A random sampling of households was done on-site using systematic sampling procedures due to the lack of official information regarding the quantity and location of households in the Wirtu Yedi kebele. Four trained data collectors who were native to the Asendabo area and were fluent in Afan Oromo conducted the data collection. The interpreters conducted the interview from the translated questionnaire and then marked an answer sheet containing the question numbers and corresponding possible choice for each question in numerical form. The interviewers were asked to circle the appropriate response on the answer sheet. The survey was conducted on 10 consecutive days from January 21, 2011 to January 31, 2011.

Data were first checked manually for completeness and then entered into Epi Info version 3.5.2 (Centers for Disease Control and Prevention (CDC), Atlanta, GA). Data were cleaned to assure completeness and consistency between questions and were presented using tables, graphs, and summary statistics.

An institutional review board (IRB) proposal was submitted to Touro University for expedited clearance. An IRB exemption was received prior to the beginning of data collection. Ethical clearance was also received from Jimma University. Data collectors informed all participants of the study about the purpose of the study in the local Afan Oromo language and verbal consent to collect and use data was obtained.

## Results

The response rate of the study was 100.0% (n = 366). Most households interviewed had at least one person who has ever had perceived symptoms of malaria (99.7%), as reported by the caretaker of the household. Of this population, 37.3% (136/365) used some sort of home care instead of or prior to seeking treatment from a healthcare facility. However, overall, 97.8% of this population eventually sought treatment for malaria at a healthcare facility. Those people who sought treatment at a facility (63.2%) did so in one day or less, 21.9% sought treatment between one to two days, 12.0% sought treatment between two to three days, and 2.9% of the people sought treatment in more than three days. Of individuals sick with malaria less than or equal to five years of age, 65.2% were taken to the healthcare institution in one day or less. Of those individuals older than five years of age, 63.2% were taken to the healthcare facility in one day or less. Of the persons sick with malaria and who received home treatment, 64.0% were younger than 10 years old. Of the total population interviewed, 2.5% (9/365) had someone in their household die of malaria.

As seen in Table 1, the sociodemographic characteristics of those caregivers who chose home treatment prior to or instead of seeking care at a healthcare facility are as follows: 45.4% of study participants were males and 54.6% were females, the majority of caretakers (33.8%) were between 15 and 25 years old, 77.3% of the caretakers were Muslim, Aromic was the most prevalent ethnicity (77.1%) among the caretakers, the majority of the caretakers (44.4%) were unable to read or write with the next most common response (14.8%) being that they were able to read but did not have any formal schooling, the median household income reported was 400 Birr per month with 300 Birr per month as the mode, and 51.5% of caretakers reported being in a polygamous marriage and 11.5% in a monogamous marriage.

**Table 1:** Sociodemographic Characteristics of the Population of Asendabo *$1.00 = 16.5 Birr

Variable	n	%
Sex		
Male	59	45.4
Female	71	54.6
Age (years)		
< 15	2	1.5
15 – 24	46	33.8
25 – 34	31	22.8
35 – 44	21	15.4
45 – 55	9	6.6
> 54	22	16.1
Religion		
Muslim	102	77.3
Orthodox Christian	28	21.2
Pen	2	1.5
Ethnicity		
Aromic	101	77.1
Amharic	12	9.2
Yem	11	8.4
Gurage	7	5.3
Education		
Unable to read and write	60	44.4
Able to read and write	20	14.8
Grade 1 – 3	8	5.9
Grade 4 – 6	14	10.2
Grade 7 – 8	11	8.1
Grade 9 – 10	18	13.3
Grade 11 – 12	2	1.5
Grade 12+	2	1.5
Monthly income (Birr*)		
< 100	2	2.2
100 – 199	21	15.4
200 – 500	55	40.4
> 500	52	38.2
Marital status		
Married (monogamous)	15	11.5
Married (polygamous)	67	51.5
Single	26	20
Widowed	17	31.1
Divorced	4	3.1

All reported home treatments within the study can be found in Table 2. Of the modern anti-malarial medications currently prescribed in the Asendabo region, the most commonly used prior to seeking treatment at the healthcare facility was Coartem, with 10.1% of the population having reported its use, 7.9% reported using chloroquine, and 0.3% reported using quinine prior to seeking professional care. Of people who used Coartem prior to going to the healthcare facility, 94.4% were able to correctly describe its administration regimen. Ninety percent of people who took chloroquine were able to correctly describe its administration regimen. The one person who used quinine was also able to correctly describe the administration regimen. Other modern medications found to be in use in the community were Paracetamol with 7.1% of caregivers having reported its use and Fansidar with 1.1% of caregivers having reported its use. As seen in Table 3, the most common source to obtain modern medications was reported to be a drug outlet (85.6%).

**Table 2:** Types of Home Treatments for Malaria (N = 365)

	n	%
Modern Drugs		
Coartem	37	10.1
Chloroquine	29	7.9
Paracetamol	26	7.1
Fansidar	4	1.1
Quinine	1	0.3
Non-pharmacological		
Ritual	18	4.9
Sponge-bathing	8	2.2
Exercise	2	0.5
Holy water	1	0.3
Herbs		
Garlic	45	12.3
Ginger	35	9.6
Harmaguse	14	3.8
Onion	7	1.9
Feto	7	1,9
Sunko	3	0.8
Damekese	2	0.5
Ganper	1	0.3
Ibicha	1	0.3
Yebudamehanit	1	0.3

**Table 3:** Reported Sources of Modern Medications

Source	n	%
Drug outlet	89	85.6
Shop	4	3.8
Past prescription leftover	1	1.0
Market	7	6.7
Neighbors	3	2.9

The most common herbs used in the community were garlic (12.3%), ginger (9.6%), and harmaguse (3.8%). Other herbs used in home treatment of malaria were damekese, feto, ganper, ibicha, onion, sunko (or habish), and one person reported using yebudamedihanit (see Appendix B for a description of treatments and their use). Of the non-pharmacological home treatments most commonly used was a religious ritual at 4.9% prevalence; others were exercise, bathing, and use of holy water.

As seen in Table 4, the reason most commonly reported for having chosen home treatment was that the healthcare facility was too far away (74.3%). The next most common reasons were that the severity of illness was too low (41.2%), the caretaker was not able to afford modern healthcare (22.1%), and to save time (21.3%). Other reasons were expecting no more benefit from going to the healthcare facility than from home treatment (8.8%) and wariness about anti-malarial drug side effects (1.5%).

**Table 4:** Reasons for Choosing Home Treatment ^†^Percentages do not add up to 100 due to multiple responses

Reasons given	n	%†
Cannot afford modern medicine	30	22.1
Low severity of illness	56	41.2
To save time	29	21.3
Health facility too far away	101	74.3
Expect no/less benefit from modern healthcare	12	8.8
Side effects of antimalarial drugs	2	1.5

**Figure 1:**
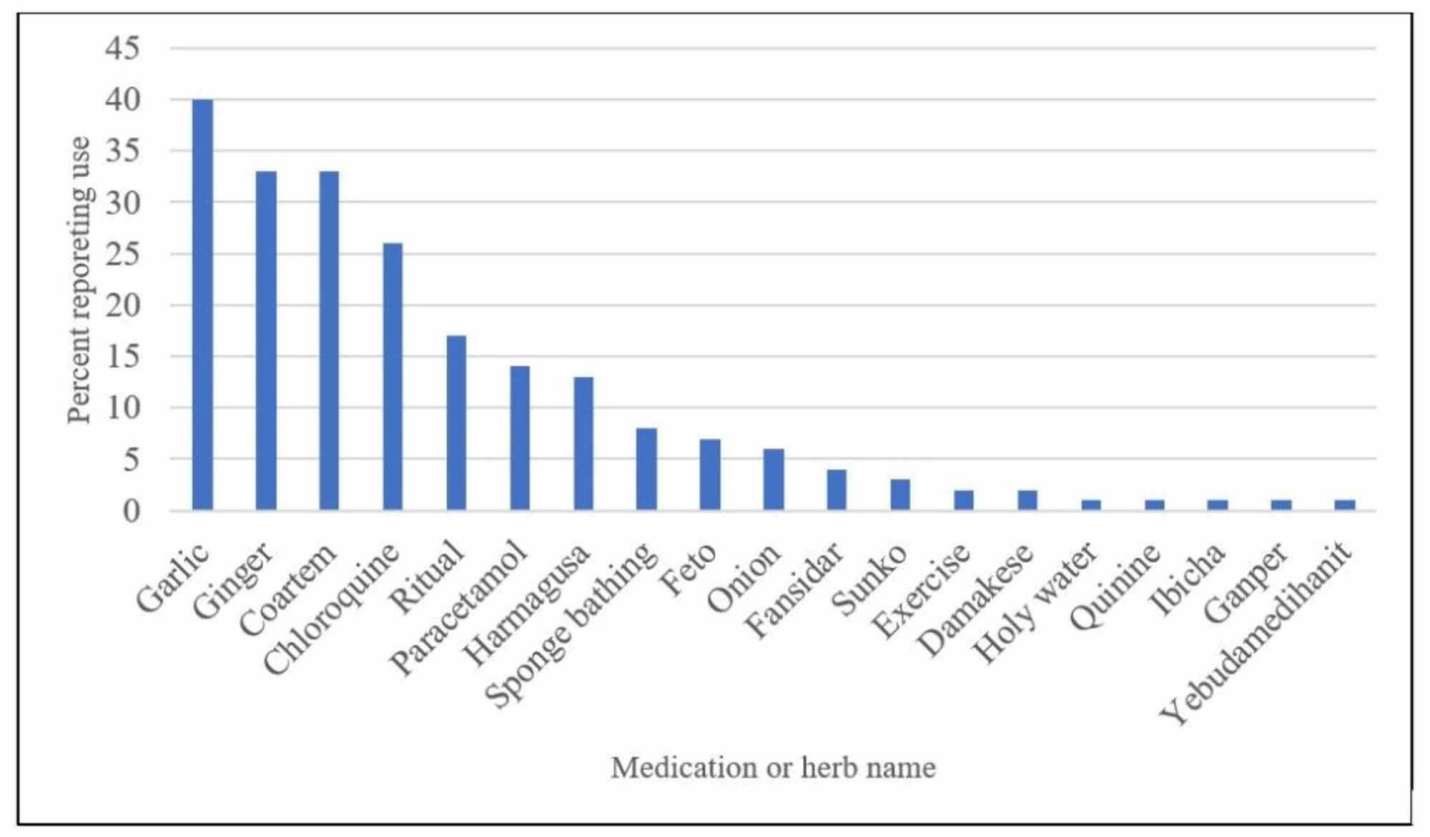
The prevalence of treatments used by caretakers who reported improvement in the patient's condition

**Figure 2:**
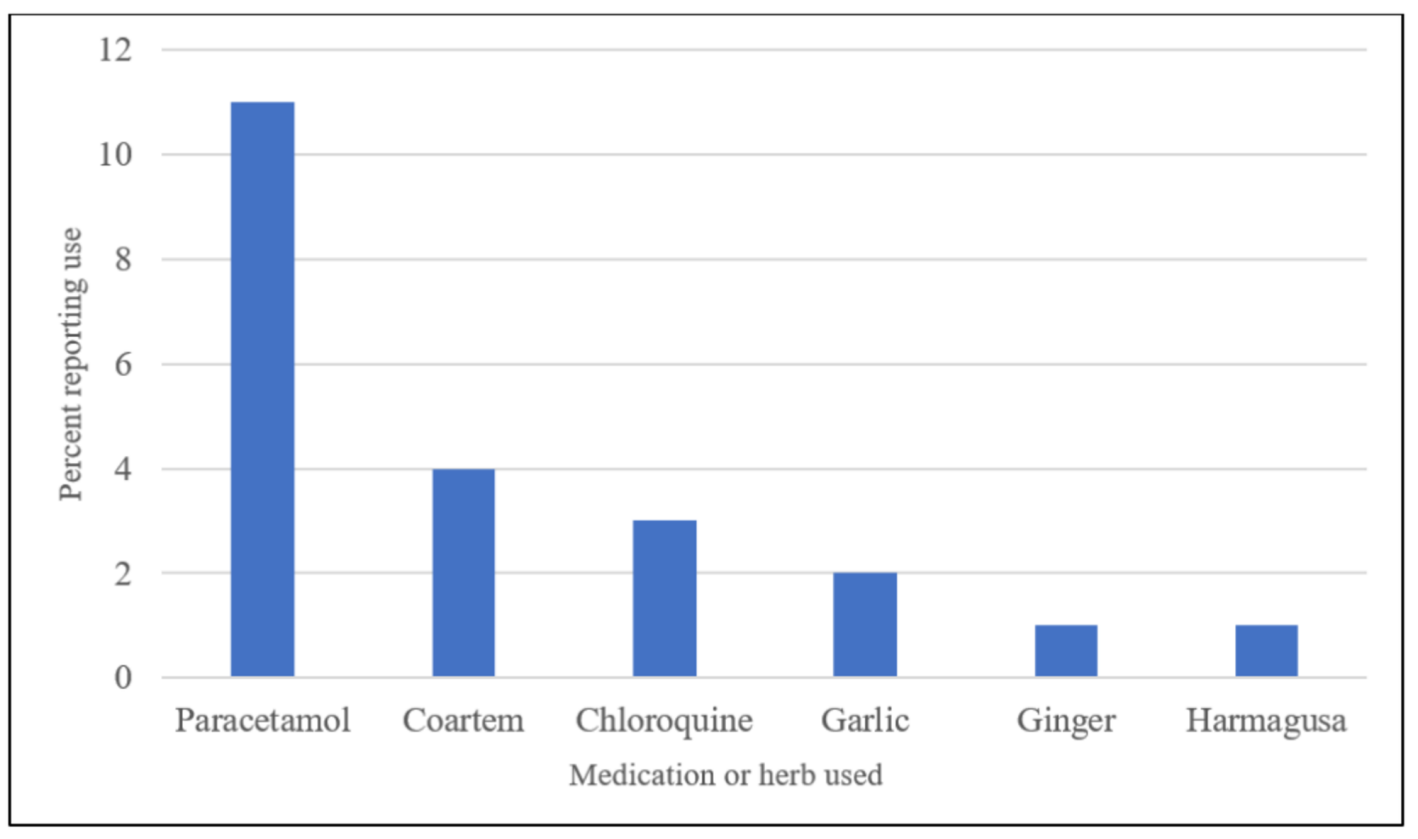
The prevalence of treatments used by caretakers who reported worsening in the patient's condition

The majority (85.1%) of caretakers reported that the home treatment they administered improved the patient’s condition, 2.2% reported no change in the patient’s condition, and 12.5% reported a worsening of the patient’s condition. Of the people who reported improvement in the patient's condition, the most commonly used modern medications used were Coartem (28.9%) and chloroquine (22.8%), and the most commonly used herb was garlic (35.1%) (Figure 1). Of the three caretakers who reported no change in the patient's condition, the only modern medication used was Paracetamol (33.3% or 1/3). Of the 17 caretakers who reported a worsening in the patient's condition, the most commonly used modern medication was Paracetamol (64.7% or 11/17), three caretakers reported using chloroquine, and four reported using Coartem (Figure 2). All individuals using the modern anti-malarial drugs who reported worsening in the patient's condition were aware of the proper administration regimen for each of those medications.

## Discussion

One of the key aspects of an effective healthcare system is one that addresses the apparent barriers that develop which restrict patients from having access to care. Within the town of Asendabo in Ethiopia, many barriers were found that ultimately affected the treatment-seeking behaviors of the community. A previous study done in Asendabo identified some of these barriers [5]. It is likely that the more significant the barrier, the higher the urge for the population to seek alternatives to modern healthcare. This study found that the prevalence of self-medication in Asendabo across all kebeles was 39.2%. We found that the prevalence of self-medication for malaria, specifically in the Wirtu Yedi kabele, was 37.3%.

Almost all people eventually sought treatment for malaria at the healthcare facility. More than half did so in less than one day. This shows the community knowledge and awareness of the potential seriousness of the condition and, ultimately, the community relying on modern healthcare as its safety net. This places more responsibility on the healthcare system to provide adequate services. Our study showed that when self-treatment was used, there was a wide array of self-medication practices used, including modern medications not prescribed by a healthcare professional, herbs, and non-pharmacological measures.

The more commonly used medications were chloroquine and Coartem. Most reported obtaining these medications at a drug outlet store without a prescription and prior to seeking care at a health center. These drugs are easily accessible due to the weak enforcement of regulations with respect to drug distribution and sales. Despite their independent use of prescription medication, perhaps due to high awareness of malarial management, almost all caretakers were able to identify the proper administration schedule. They also commonly reported a self-assessed improvement in patient outcomes as a result of home treatment. Another commonly used modern medication is Paracetamol. This analgesic was mainly used to treat the prodromal period of malaria. Of some concern is that a large proportion of the population who reported worsening symptoms in the patient outcome had used Paracetamol. This could be due to the community’s misconception that this medication does not treat the underlying etiology but is meant for only symptomatic relief. This medication offers a false sense of security by temporally alleviating the symptoms of malaria without addressing the underlying cause, thereby allowing the disease to progress. Community education regarding the proper use of Paracetamol could help to alleviate the misuse of this medication. Of the various herbs reported that were used to self-treat malaria, the most commonly used were garlic, ginger, and harmaguse. The use of herbs was found to be less common than the use of modern medication. Of the non-pharmacological interventions, rituals were the most prevalent. This could perhaps be due to every respondent identified themselves with some sort of religious affiliation which shows the importance of religion in the community.

When considering some of the reasons why people chose home care, the most common reason was the health facility was perceived as too far away. Geographically, this kebele is only about 20 - 30 minutes walking distance to the nearest healthcare center. Thus, the apparent barrier might not be the actual physical distance but instead might be the difficulty in getting the bedridden patient from the household to the health center. This apparent barrier needs further investigation but may support the development or introduction of a structured emergent ambulatory service. Other noteworthy barriers as to why caregivers chose home care included low severity of illness and to save time and/or money. These barriers are consistent with those cited in previous studies [7].

There were limitations to our study that make the interpretation and generalizability of results more difficult. Our study collected information regarding the most recent case of malaria in each household. However, we did not inquire as to the amount of time since the last episode of illness. The amount of time elapsed since the last illness in the household could have been highly variable and possibly lengthy. This naturally introduces recall bias into the interviewees’ responses; information regarding the most recent cases presumably being the most accurate. Another limitation stems from the fact that anti-malarial medications are intended to be prescribed by the healthcare worker and are not meant for personal administration. This adds to the possibility that responders were more likely to provide socially acceptable responses regarding the use of anti-malarial drugs. Also, variable interviewer styles and techniques from each individual data collector could have issued variable responses to identical questions. We are also unable to make statements regarding the validity and reliability of the results due to the methodology and design of the study. Another category of limitations is the possible misinterpretation of the intended meaning of the specific questions on the data collection instrument. The specific problem encountered with the questionnaire was the misinterpretation by the responders of question number 9 to mean “how long did it take you to get to the healthcare center?” Another problem noted was the fact that some respondents answered that they did not use any “treatment” for malaria prior to seeking care at the healthcare center and yet responded that they used herbs or other non-pharmacological measures. This may be due to the respondents’ perception that the term “treatment” only refers to modern medications. Issuing a pilot study to test the data collection instrument prior to starting the study, as well as to conduct focus groups in order to make the specific questions more culturally specific, could have minimized the impact of this limitation. These precautions were not taken due to time constraints.

## Conclusions

This study has identified that the Wirtu Yedi kebele relies heavily on modern medicine to treat illness. Although the proximity of the health center to this kebele is relatively close, there still seems to be some apparent barriers that prevent adequate access. Regarding this, further studies should concentrate on identifying the community's perceived barriers of access to care. One potentially significant investigation should look into a needs assessment of the development of structured emergent ambulatory services for the community. This study also identified the most common modern medications used at home to treat illness. The data showed that many caregivers have easy obtainable access to modern medications, despite bypassing the healthcare facility system. This can lead to dangerous outcomes from side effects and/or drug interactions if not managed properly by a healthcare professional. It is suggested that the community look into developing a tighter regulation on dispensing medications without a prescription. Additionally, the community commonly used other treatments that were not anti-malaria-based but were perceived by the caregiver to help aid in the patient outcome. Our suggestion for further studies would be to investigate the actual effectiveness of these home therapies.

## Appendices

Appendix A: Structured Questionaire

SELF-MEDICATION PRACTICES SURVEY

Questionnaire to be administered to the head of households of residences who reside in Assendabo to assess current malaria self-treatment practices.

Instruction to the interviewer:

This questionnaire targets residences of Assendabo that live in Wirtu Yedi kebele only. Ask the head of the household or other available members of the household and get consent to administer the questionnaire. For each question, circle the numbered code of each response in the right-hand column or fill in the information as indicated.

STOP should indicate proceed to next house.

Statement of Informed Consent

Dear participant,

You have been identified as a relevant respondent for this study. We are Touro University students working with Jimma University to find out what people in the local area use to treat malaria in their homes. The questionnaire contains 23 questions and the interview will take approximately 20 minutes. The information you provide is confidential and will only be used to assess malaria treatment practices in this community.

1. House number

2. Date of interview

3. Data collector: Name                            , Signature                                         

4. Do you know what malaria is?

Yes……………………..1

No………………………2

5. Have you or anyone else in your household ever had malaria?

Yes………………………1

No……………………….2 --> STOP

6. What was the age of the person sick with malaria?

Age_____________

Or

SELF

7. Has anyone in your family ever died of malaria?

Yes………………………1

No……………………….2

8. Did you go to any healthcare institution to seek treatment for malaria?

Yes………………………1 --> go to question 9

No ………………………2 --> go to question 11

9. How long did it take you to seek care at the health center? 

_________________

10. Did you try any treatment before going to the health center?

Yes ……………………..1 --> go to question 12

No ………………………2 --> STOP

11. Did you try any treatment for malaria?

Yes………………………1 --> go to question 12

No ………………………2 --> STOP

12. What home care did you use?

Herbs

Ginger…………………………………..1

Garlic…………………………………….2

Harmagusa……………………………3

Other ___________________...4

Non-Pharmalogical

Holy water…………………………….5

Sponge bathing……………………..6

Exercise…………………………………7

Rituals……………………………………8

Other __________________.....9

Modern drugs

Chloroquine……….10 --> go to question 11

Coartem…………..…11--> go to question 12

Quinine……………….12--> go to question 13

Other ______________...........13

Anything else?__________________________

13. How did you take Chloroquine?

(correct regimen: single dose)

Correct regimen………………………….1

Incorrect regimen……………………….2

14. How did you take Coartem?

(correct regimen: 1st day every 8 hr, 2nd day every 12 hr, 3rd day every 12 hr)

Correct regimen………………………….1

Incorrect regimen……………………….2

15. How did you take Quinine?

(correct regimen: q8h for 5 - 7 days)

Correct regimen………………………….1

Incorrect regimen……………………….2

16. If took Chloroquine, Coartem or Quinine ask:

Where did you obtain the medication?

Drug outlet………………………………1

Private clinic……………………………..2

Shop…………………….........……………3

Past prescription leftover…….....4

Market………………………….........……5

Neighbors………………........…………6

Other__________________....7

17. Do you believe the treatment you administered…

Improved patient condition…….....1

Didn’t change patient condition..2

Worsened patient condition…..….3

18. What influenced your decision most to use self-medication?

Cannot afford modern health care….1

Low severity of illness ……............…….2

To save time………………….................……3

Health facility too far away…...........….4

The expectation of less/no benefit from modern health care……..5

Side effects of antimalarial drugs…..6

Other___________________

19. Age (of person administering treatment)

20. Sex (of person administering treatment)

Male……………………………….….1

Female ……………………………..2

21. Religion (of the person administering treatment)

Muslim………………….……………1

Orthodox…………………………..2

Pent……………………….………….3

Other______________....4

22. Language

Aromic……………………1

Ahmaric………………….2

Yem………………………..3

Gurage…….…………….4

Other_________.....5

23. Educational status (of the person administering treatment)

Unable to read or write….……..1

Able to read and write………….2

Grade 1-3……………….....…………..3

Grade 4-6…………………...………..4

Grade 7-8 ………………...………….5

Grade 9-10……………………………6

Grade 11-12 ……………....…………7

Grade 12+…………………………….8

24. Household income (indicate amount per year OR per month)

Birr_____________________per year

or

Birr___________________per month

25. Marital status (of person administering treatment)

Married (monogomous)………1

Married (polygamous)…….….2

Single…………………………………..3

Widowed………………………….…4

Divorced……………………………..5

**Appendix B: Glossary (T. Mekonnen, personal communication, February 31, 2011)**

1. Ginger - This is crushed and then mixed with water. It is then boiled and sugar is added and is drunk as a tea preparation. In some households, ginger, garlic, and salt are sometimes crushed together and consumed with food at higher than usual amounts.

2. Harmagusa - These are picked green leaves that are crushed. As they are crushed, a precipitate is squeezed out of the plant and is prepared into a drink.

3. Ibicha - Type of plant that is crushed and squeezed with water. The juice from the plant and water is made into a drink.

4. Damekese - Good smelling green leaves that are crushed and form a precipitate. Both remaining crushed leaves and precipitate is usually put in the nose for rhinorrhea. Also used for symptoms of the common cold, such as sneezing and headache.

5. Sunko (Habish) - Crushed cereal from oats that is mixed with water and sugar. It is put in the fridge and then drunken. Used for rehydration and food supplementation.

6. Yebudamidihamt - Medication used by the “local healer” to attack various symptoms that are believed to be caused by an evil spirit. The preparation is only known by the local healer but is usually a combination of various plants.

7. Fansidar - Type of modern medication that was once used for malaria caused by P. falciform. This medication was discontinued three years ago due to resistance in the local area.

8. Feto - cereal that is crushed finely and put into water until porridge-like consistency results. It is usually consumed with other food. Used for common cold symptoms, as well as a decongestant.

9. Paracetamol: This drug goes by the name Normacid (magnesium trisilicate) but prescribed by the name Paracetamol. This salicylate derivative is commonly prescribed for headache and fever and is sometimes used for symptoms of malaria.

10. Coartem 20/120 (artemether/lumefantrine): A modern anti-malarial agent currently prescribed in Ethiopia. The medication is free when prescribed for confirmed cases of malaria. The administration regimen is 4 tabs twice per day for three days for patients over 35 kg and 3 tabs twice per day for three days for patients below 35 kg and above 25 kg.
